# PRDM15 regulates differentiation and proliferation of hematopoietic stem cells

**DOI:** 10.21203/rs.3.rs-7956606/v1

**Published:** 2025-11-03

**Authors:** Ernesto Guccione, Nesteene Param, Alex Rialdi, Nayeli Guiterrez Trejo, Max Fortescue-Poole, Ke Wang, Habiba Zorgati, Daniel Lozano-Ojalvo, Magan Schwarz, Francesco Cantatore, Frank Fonseca, Kensey Portman, Kevin Mohammed, David Dominguez-sola, Slim Mzoughi, Elvin Wagenblast

**Affiliations:** Icahn School of Medicine at Mount Sinai; Icahn School of Medicine at Mount Sinai; Icahn School of Medicine; Icahn School of Medicine at Mount Sinai; Icahn School of Medicine at Mount Sinai; Icahn School of Medicine at Mount Sinai; Icahn School of Medicine at Mount Sinai; INSTITUTO DE INVESTIGACION EN CIENCIAS DE LA ALIMENTACION; Icahn School of Medicine at Mount Sinai; Icahn School of Medicine at Mount Sinai; Icahn School of Medicine at Mount Sinai; Icahn School of Medicine at Mount Sinai; Icahn School of Medicine at Mount Sinai; Icahn School of Medicine at Mount Sinai; Mt Sinai; Icahn School of Medicine at Mount Sinai

## Abstract

Hematopoietic stem cells (HSCs) sustain lifelong hematopoiesis through a tightly regulated balance of self-renewal, proliferation and differentiation, particularly under stress conditions.

Here, we identify PRDM15, a transcription factor with well described roles in early embryonic development, as a crucial regulator of hematopoiesis during stress responses. While PRDM15 deletion is tolerated at steady state in adult hematopoiesis, its absence severely impairs bone marrow reconstitution following transplantation, causing sustained reduction in bone marrow cellularity and differentiation blocks. Notably, PRDM15-deleted bone marrow exhibited an accumulation of stem and progenitor cells, indicating a block in lineage differentiation. Furthermore, in competitive transplantation assays, PRDM15-deficient cells were unable to compete with wild-type counterparts, demonstrating a profound loss of fitness.

Transcriptomic and epigenomic analyses reveal PRDM15 as a critical regulator of differentiation and proliferation of HSCs. Mechanistically, PRDM15 directly regulates the expression of several key genes involved in proliferation and differentiation pathways, including the transcription factor *Cux1*. *Cux1* overexpression partially rescues colony-forming ability of PRDM15-deficient HSCs.

These findings establish PRDM15 as a pivotal stress-responsive regulator of HSC differentiation and survival, with implications for therapeutic modulation of hematopoiesis.

## INTRODUCTION

The tight regulation of HSCs self-renewal versus differentiation trajectories is driven by cytokine signaling^1–6^, transcriptional-^7–10^ and metabolic- regulation^11,12^ and is essential to ensure the production of hematopoietic cells throughout an animal’s lifespan. This regulation is dose- and context-dependent, and we have yet to identify all factors involved in this central process^13^.

Several members of the PRDM (PRDI-BF1 and RIZ homology domain-containing) family have been characterized as key transcriptional regulators of hematopoiesis^14^. For instance, PRDM3 is essential for the maintenance of both fetal and adult HSCs, albeit not lineage commitment^15^. PRDM16 is a key regulator of early hematopoiesis by regulating the cell cycle and the levels of reactive oxygen species^16,17^. Downstream lineage commitment is also influenced by PRDMs. PRDM2, typically expressed in mature myeloid and CD34 + cells, acts as a tumor suppressor in chronic myeloid leukemia^18^. Another family member, PRDM1 (also known as BLIMP1), regulates terminal differentiation of B cells and T cells^19–21^.

PRDM15 has been initially characterized as an essential transcription factor both for the maintenance of embryonic stem cells naïve state by modulating the MAPK-ERK and WNT pathway^22^ and for developmental patterning via regulation of NOTCH and WNT/PCP pathways^23^. Notably, while PRDM15 is critical for embryogenesis, with deletion leading to embryonic lethality between E12.5 - E14.5 in the mouse^23^, its presence is dispensable in adult mice maintained in a sterile environment^24^.

While PRDM15 has yet not been directly implicated in hematopoiesis, it was additionally characterized as a key factor promoting MYC-driven B cell lymphomagenesis. In B cell lymphomas, PRDM15 is required to rewire their metabolism by regulating the mTOR/PI3K pathway, and targeted PRDM15 depletion in lymphoma cells results in tumor regression^24^.

Given the link between mTOR/PI3K and normal hematopoiesis^25^, we decided to specifically explore PRDM15’s role in conditions in which HSCs or progenitors would be forced to reconstitute the entire blood system^26,27^. To investigate this, we first deleted PRDM15 in isolated fetal liver CD34 + HSPCs, prior to transplantation into sub-lethally irradiated mice. Loss of PRDM15 led to decrease bone marrow engraftment. Next, to gain mechanistic insights we switched to murine experimental models. We transplanted PRDM15^fl/fl^;R26^cre/cre^ and PRDM15^fl/fl^;Vav^cre^ bone marrow into lethally irradiated wild-type recipients. In the R26^cre/cre^ model, tamoxifen-induced PRDM15 deletion led to a sustained reduction in bone marrow cellularity from week 1 through week 28. Similarly, PRDM15 loss in the Vav^cre^ model resulted in decreased CD45 + cells and reduced mature hematopoietic populations. Notably, PRDM15-deleted bone marrow exhibited an accumulation of stem and progenitor cells without changes in proliferation, indicating a block in lineage differentiation. Furthermore, in competitive transplantation assays, PRDM15-deficient cells were unable to compete with wild-type counterparts, demonstrating a functional disadvantage. Transcriptomic profiling of hematopoietic stem and progenitor cells revealed downregulation of differentiation and proliferation pathways. *Cux1*, a directly bound and regulated PRDM15-target, is a transcription factor that regulates cell cycle progression^28 29^, DNA damage^30^ and chromosomal stability^31^. *Cux1* is also known as a key regulator of HSC quiescence and proliferation^32–34^. These findings establish PRDM15 as a critical regulator of hematopoiesis under proliferative stress, partially acting through transcriptional control of *Cux1* in hematopoietic stem and progenitor cells.

## RESULTS

### PRDM15 deletion in human HSPCs decreases bone marrow engraftment.

**Figure 1:**
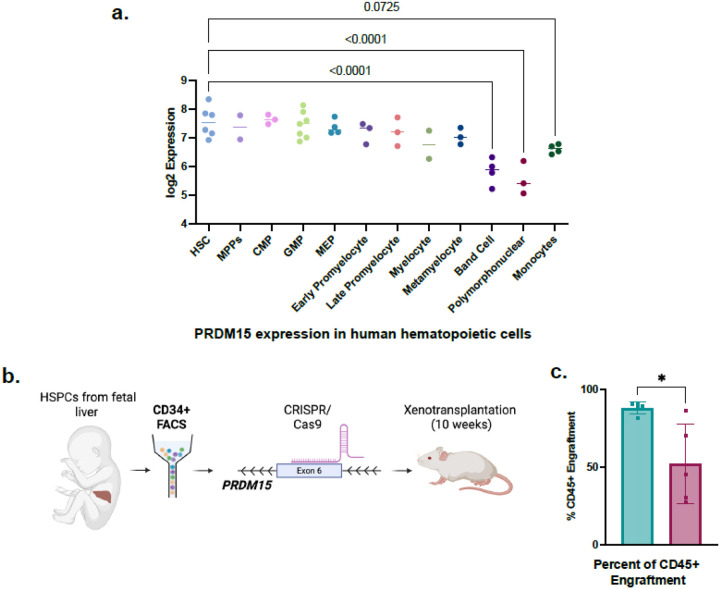
PRDM15 deletion in human HSPCs decreases bone marrow engraftment. a. PRDM15 expression in human hematopoietic cells derived from bloodspot.eu b. Schematic for human HSPC PRDM15 deletion and xenotransplantation. c. Percent of CD45+ engraftment in PRDM15 knockout compared to control. Unpaired t test. Error bar represent SEM. * p ≤ 0.05

To investigate the role of PRDM15 in human hematopoiesis, we first examined its expression in HSCs and mature lineages^35^. PRDM15 is highly expressed in human hematopoietic cells, with increased expression in HSPCs compared to mature cells, like band cells, polymorphonuclear cells and monocytes (Fig. 1a). While no changes in hematopoiesis were explored or reported in human with biallelic *PRDM15* loss-of-function variants, families with such mutations have been identified with Galloway-Mowat syndrome (GAMOS) or holoprosencephaly (HPE), demonstrating that loss of PRDM15 disrupts normal renal and brain development^23,36^.

To determine if PRDM15 deletion alters HSPCs function, we isolated fetal liver CD34 + HSPCs, induced CRISPR/Cas9 mediated PRDM15 deletion and transplanted cells into sub-lethally irradiated NSG mice. Engraftment efficiency was analyzed at week 10 post-transplant (Fig. 1b). When PRDM15 is deleted in HSPCs, CD45 + engraftment is significantly decreased compared to wildtype control (Fig. 1c). PRDM15 deletion efficiency was 54.7% effective prior to xenotransplantation (**Supplementary Fig. 1a**). Levels of PRDM15 deletion decreased to an average of 21.46% suggesting that residual wildtype cells out-compete PRDM15 deficient HSPCS (**Supplementary Fig. 1b**). These findings show that PRDM15 regulates bone marrow engraftment and may play a role in hematopoietic differentiation. Having established that PRDM15 deficiency affects human hematopoietic function, we next sought to explore the underlying mechanisms of this activity using murine models.

### PRDM15 is required for bone marrow reconstitution and stem cell differentiation.

To investigate the role of PRDM15 in murine hematopoiesis, we first examined its expression in HSCs and mature lineages. PRDM15 was ubiquitously expressed across all hematopoietic stem and progenitor cells (HSPCs) and mature counterparts with notably decreased expression in monocytes (**Supplementary Fig. 2a**).

**Figure 2:**
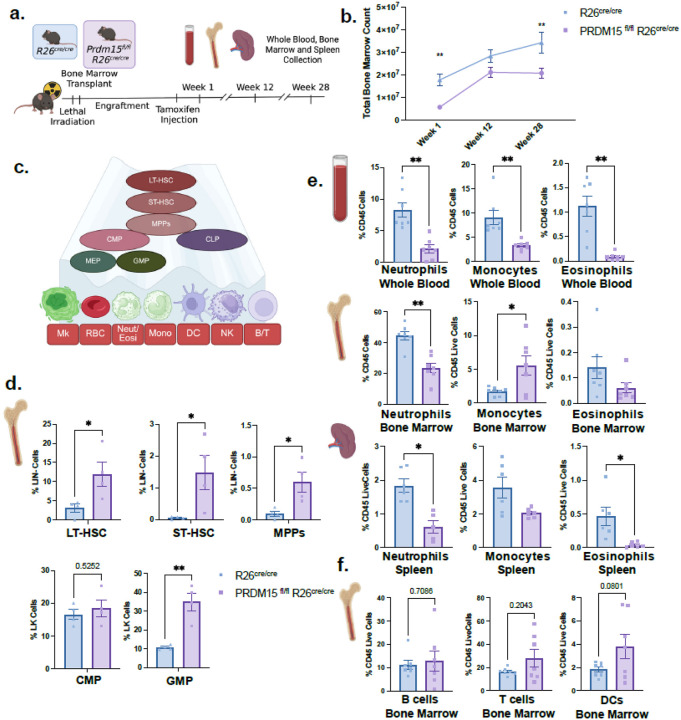
PRDM15 is expressed in hematopoietic stem and progenitor cells (HSPCs) and is required for complete bone marrow reconstitution and stem cell differentiation. a. Wildtype CD45.1, 8–10 weeks old female (Host) are lethally irradiated (1050 cGy). PRDM15fl/fl R26cre/cre or R26cre/cre females (Donor) bone marrow is transplanted into host animals retroorbitally. 1 month post transplantation, tamoxifen is injected into host animals to induce recombination in donor bone marrow cells. Blood, spleen and bone marrow were harvested in three timepoints, 1 week, 12 weeks and 28 weeks post tamoxifen injection. b. Bone marrow counts of three timepoints. 2-way anova. c. Schematic for hematopoietic development from hematopoietic stem cells (HSCs). LT-HSC (long term HSC), ST-HSC (short-term HSC), MPPs (multipotent progenitors), CMP (common myeliod progenitor), GMP (granulocyte-monocyte progenitor), MEP (megakaryocyte-erythroid progenitor), CLP (common lymphoid progenitor), Mk (megakaryocyte), RBC (red blood cell), Neut/Eosi (Neutrophils and Eosinophils), Mono (monocytes), DC (dendritic cell), and NK (natural killer cells). d. Bone marrow cells from reconstituted animals one week post tamoxifen injection. LT-HSC, ST-HSC, MPPs, CMP and GMP. Unpaired t-test. e. Percentage of mature granulocytes and monocytes of whole blood, spleen, and bone marrow from PRDM15fl/fl R26cre/cre or R26cre/cre reconstituted mice. f. Percentage of B cells, T cells and DCs in bone marrow cells, 1 week post tamoxifen. Unpaired t-test. Error bars represent standard error of the mean (SEM). * p ≤ 0.05, ** p ≤ 0.01, *** p ≤ 0.001, **** p ≤ 0.0001.

To directly asses the functional relevance of PRDM15 in hematopoiesis, we generated tamoxifen-inducible PRDM15^fl/fl^;R26^cre/cre^ mice and transplanted their bone marrow into lethally irradiated CD45.1 recipients (Fig. 2a). Following tamoxifen administration post engraftment, PRDM15 deletion resulted in significant and sustained reduction in total bone marrow cellularity from 1 to 28 weeks (Fig. 2b), suggesting that PRDM15 is required to maintain normal hematopoietic output.

To determine whether HSPCs were affected by PRDM15 loss, we performed flow cytometry on lineagenegative (Lin-) cells 1 week post tamoxifen injection. PRDM15 deletion caused marked accumulation of LT-HSC, ST-HSC, MPPs, and GMPs (Fig. 2c–d), with no significant changes in CMP, MEP and CLP (Fig. 2d and **Supplementary Fig. 2b**). Mature hematopoietic cells, especially granulocytes and monocytes, showed significant reductions in peripheral blood, spleen, and bone marrow (Fig. 2e) whereas the lymphoid and dendritic cell compartment remained unaffected (Fig. 2f). The accumulation of HSPCs accompanied by a reduction in mature cells suggests a differentiation block, particularly within the GMP population. Gating strategy is summarized in **Supplementary Fig. 2c-d.** By 12 weeks post tamoxifen injection, progenitor cell frequencies returned to baseline, however reduction in the B cell and monocyte populations reflect delayed lymphoid differentiation defects (**Supplementary Fig. 2e-f**). Together, these findings demonstrate that PRDM15 is essential for proper HSPC differentiation and is required for both myeloid and lymphoid lineage generation.

### PRDM15 deletion impairs immune recovery after proliferative stress.

**Figure 3:**
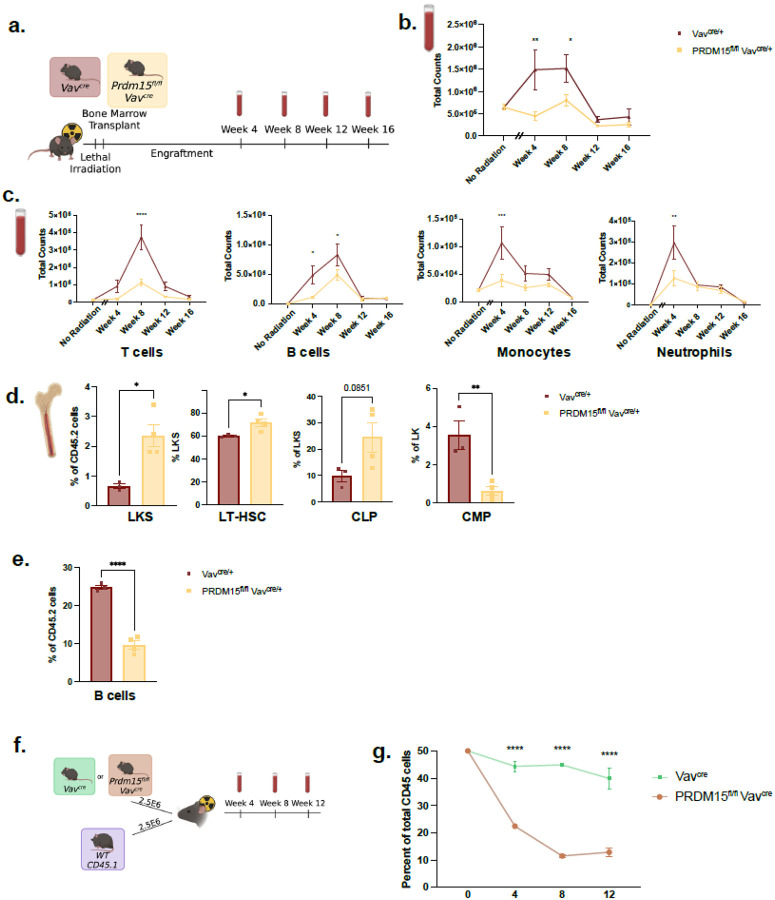
Hematopoietic cell-specific deletion of PRDM15 causes a severe defect in bone marrow cell production and impairs maturation of all blood lineages. a. Wildtype CD45.1, 8–10 weeks old female (Host) are lethally irradiated (1050 cGy). PRDM15fl/fl Vavcre or Vavcre 6–8 week old females (Donor) bone marrow is transplanted into host animals retro-orbitally b. Total counts of CD45.2 positive cells in blood cells. Two-Way Anova. c. Total counts of whole blood at Week 4, 8, 12 and 16 in female host and donor mice. Two-Way Anova. d. Percentage of LKS cells in CD45.2+ cells. Percentage of LT-HSC and CLP in LKS positive cells. Percentage of CMP in LK positive cells. e. Percent of B cells in CD45.2 positive cells. Unpaired t test. f. Competition study using PRDM15 fl/fl Vavcre or Vavcre (CD45.2 Donor) and Wildtype (CD45.1 Donor) transplanted into Wildtype (CD45.1/CD45.2 Host). g. Blood samples analyzed for flow at Week 4, 8, and 12. Two-Way Anova. Error bars represent SEM. * p ≤ 0.05, ** p ≤ 0.01, *** p ≤ 0.001, **** p ≤ 0.0001.

Building on these observations, we examined the role of PRDM15 specifically in hematopoietic cells using the PRDM15^fl/fl^;Vav^cre^ model, targeting PRDM15 deletion specifically to hematopoietic cells (Fig. 3a and **Supplementary Fig. 3a**). Under steady-state conditions, these mice displayed near-normal hematopoiesis, indicating PRDM15 is dispensable under homeostatic conditions (**Supplementary Fig. 3b**). However, following transplantation into irradiated hosts, PRDM15^fl/fl^;Vav^cre^ exhibited a pronounced inability to repopulate mature immune lineages in CD45.1 hosts 4–8 weeks post transplantation compared to wildtype Vav^cre^ (Fig. 3b). Specifically, Monocytes, T- and B-cells were significantly reduced at 4–8 weeks post-transplant, while neutrophils returned to baseline levels at week 8 post-transplant (Fig. 3c). This suggest that induced proliferative stress, which we mimicked here through bone marrow transplantation, enhances PRDM15 defects. In this model, proliferative stress is sustained for 4–8 weeks post transplantation and resolves by around 12–16 weeks.

In the bone marrow compartment, we enriched HSPCs by sorting for c-Kit positivity. Since c-Kit expression decreases during maturation, c-Kit + and c-Kit- fractions were analysed as enriched immature and mature populations respectively^37^. At 4 weeks post-transplant, PRDM15^fl/fl^;Vav^cre^ exhibited increased frequencies in the LKS (comprises LT-HSC, ST-HSCs and MPPs), LT-HSC and CLP, accompanied with reduced CMPs (Fig. 3d). With no significant changes in ST-HSCs, MPPs, GMP and MEPs (**Supplementary Fig. 3c**) This failure in differentiation resulted in reduced total numbers of mature cells in whole blood (Fig. 3c) and decreased B cell frequencies in the bone marrow (Fig. 3e). By 16 weeks post-transplant, when proliferative stress has subsided, the effects of PRDM15 deletion on bone marrow counts are comparable to wildtype indicating that the phenotype is specific to exposure to proliferative stress (**Supplementary Fig. 3d**).

Together, these results demonstrate that PRDM15 is required for efficient hematopoeitic regeneration under proliferative stress, acting to sustain proper HSPC differentiation and maturation.

### PRDM15 Deletion Reduces HSC Fitness Under Competitive Stress.

To assess whether the observed increase in LT-HSC reflected enhanced stem cell fitness, we performed a competitive transplantation assay, in which PRDM15^fl/fl^;Vav^cre^ (CD45.2) or Vav^cre^ (CD45.2) cells are co transplanted with wildtype (CD45.1) cells into irradiated CD45.1/CD45.2 heterozygous mice. Peripheral blood was analyzed at weeks 4, 8 and 12 post-transplant to track the changes in CD45 levels in circulation (Fig. 3f). PRDM15 deletion leads to loss of competitive repopulating ability compared to wildtype cells, shown by significantly reduced bone marrow engraftment (Fig. 3g).

These findings indicate that PRDM15 loss impairs the ability of immature hematopoietic cells to sustain competitive engraftment under stress conditions. These results highlight PRDM15 as a key regulator of hematopoietic stem cell fitness and as required for effective reconstitution.

### Transcriptomic Analyses Reveal Key Differentiation and Metabolic Pathways Regulated by PRDM15.

**Figure 4:**
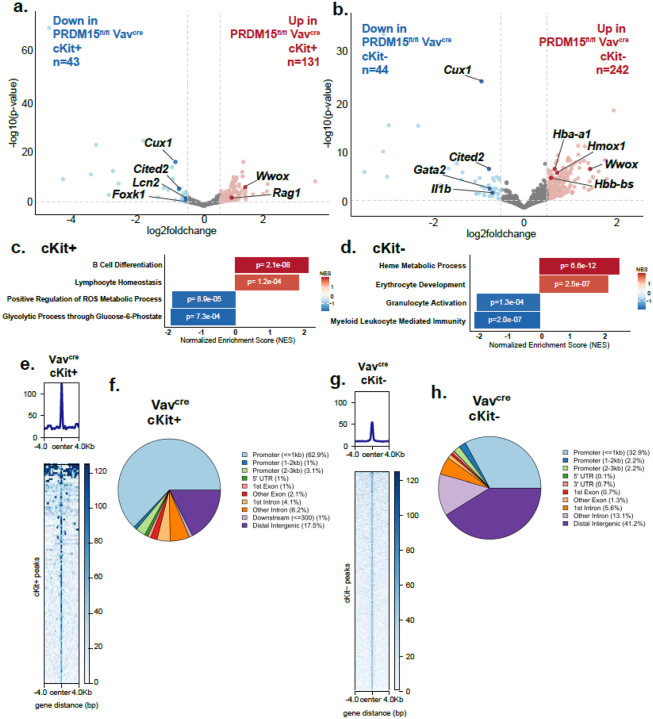
PRDM15 deletion decreases expression of genes critical for immune differentiation and metabolism a-b. Volcano plot of (a) cKit+ and (b) cKit- of differentially expressed genes after PRDM15 deletion 4 weeks post transplant injection. Adj p value >0.05 and −0.5 < log2FoldChange >0.5. c-d. Representative GSEA of PRDM15fl/fl Vavcre or Vavcre bone marrow 4 weeks post transplant in (c) cKit+ and (d) cKit-. Red depicts downregulated and blue depicts upregulated processes. Derived from Enrichr. e-f. ChIP seq in (e) cKit+ average signal (top) and density plots of PRDM15 occupancy centered on peaks (bottom) and distribution of PRDM15 peaks in relation to genes in (f) cKit+. The line plot represents the average ChIP-seq signal intensity +/− 4 kb from the peak center. g-h. ChIP seq in (g) cKit- and distribution of PRDM15 peaks in relation to genes in (h) cKit-.

PRDM15^fl/fl^;Vav^cre^ and Vav^cre^ bone marrow samples 4 weeks post transplantation were sorted into c-Kit + or c-Kit- cells, which enriched for immature or mature cells, respectively, and processed for RNA-sequencing. Transcriptomic analysis of these sorted hematopoietic populations revealed gene expression changes upon PRDM15 deletion (**Supplementary Fig. 4a**). In c-Kit + cells, PRDM15 deletion leads to 131 upregulated genes and 43 downregulated genes (Fig. 4a and **Supplementary Table S1**). Pathways associated with metabolism like glycolytic process through glucose 6-phosphate (e.g. *Foxk1)* and positive regulation of reactive oxygen species (ROS) (e.g. *Lcn2)* are downregulate and pathways associated with lymphocyte homeostasis, especially B cell differentiation (e.g. *Rag1)* are upregulated in PRDM15 depleted cells (Fig. 4c and **Supplementary Table S3**). The mature hematopoeitic cells (i.e. c-Kit-) similarly displayed extensive gene dysregulation with 242 upregulated genes and 44 downregulated genes upon PRDM15 deletion (Fig. 4b and **Supplementary Table S2**). The pathways downregulated in the mature compartment are associated with myeloid immunity and granulocyte activation (e.g. *Gata2 and Il1b)*, while the upregulated genes are associated with erythrocyte development (e.g. *Hba-a1 and Hbb-bs)* and heme metabolic process (e.g. *Hmox1)* (Fig. 4d and **Supplementary Table S4**). Together, these findings indicate that PRDM15 supports hematopoietic homeostasis by maintaining transcriptional programs required for differentiation, and metabolic balance, particularly under conditions of proliferative stress. To determine the direct targets for PRDM15 in hematopoietic cells, we performed ChIP seq (Chromatin Immunoprecipitation Sequencing) on sorted c-Kit + and c-Kit- bone marrow cells. Given that both PRDM15^fl/fl^;R26^cre/cre^ and PRDM15^fl/fl^;Vav^cre^ models displayed similar phenotypes, we used bone marrow cells derived from Vav^cre^ bone marrow cells to dissect PRDM15-dependent transcriptional regulation. We took Vav^cre^ bone marrow cells 4 weeks after transplant to replicate the PRDM15 binding activity during proliferative stress. In c-Kit + cells, PRDM15 was bound to 109 unique genomic locations, predominantly located at gene promoter regions (Fig. 4e,f and **Supplementary Table S5**). In c-Kit- cells, PRDM15 instead bound to 1396 unique peaks primarily promoters and distal intergenic regions (Fig. 4g,h and **Supplementary Table S6**).

### PRDM15 regulates HSC differentiation and proliferation partially through Cux1.

**Figure 5:**
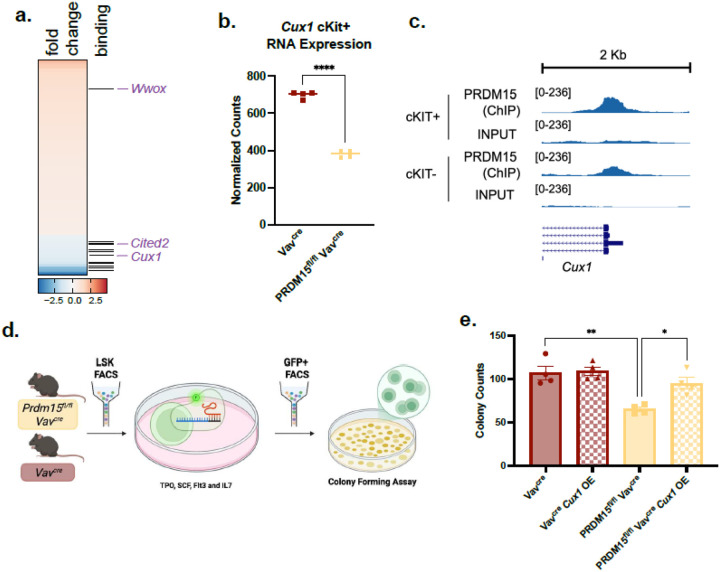
PRDM15 regulates HSC differentiation and proliferation partially through Cux1. a, Heatmap of all deregulated genes ranked from most upregulated to most downregulated after PDRM15 deletion. Black lines indicate PRDM15 binding, highlight genes noted to play a role in hematopoiesis. b. Cux1 relative RNA expression in Vavcre and PRDM15fl/fl Vavcre. c. PRDM15 ChIP enrichment peaks of Cux1 promoter in cKIT+ and cKIT- bone marrow cells. d. Schematic of selection for colony forming assay. The bone marrow cells from PRDM15fl/fl Vavcre or Vavcre were sorted for HSCs and these HSCs were plated in media containing the cytokines TPO, SCF, Flt3 and IL7. After cells are acclimated to media, HSCs were infected with Cux1 lentivirus overexpression vector with GFP reporter. Cells were then sorted for GFP positive and plated in methylcellulose plates. e. Total colony counts from controls, Vavcre and PRDM15 fl/fl Vavcre compared to Cux1 overexpression (OE) counterpart. One way anova. Error bars represent SEM. * p ≤ 0.05, ** p ≤ 0.01, *** p ≤ 0.001.

Within the bound and regulated targets, we noted genes of interest with known activity in hematopoiesis, like *Wwox, Cited2*, and *Cux1*^32–34,38–41^ (Fig. 5a and **Supplementary Table S7**). PRDM15 negatively regulates *Wwox* and PRDM15 positive regulates *Cited2* and *Cux1*. We next decided to focus on *Cux1 as* an important target due to its role in quiescence and proliferation of HSCs^32–34^. *Cux1* expression decreases after PRDM15 deletion (Fig. 5b) and PRDM15 binds strongly to the *Cux1* promoter region which was verified by ChIP qPCR with positive and negative controls. (Fig. 5c and **Supplementary Fig. 5a-b**).

To test if PRDM15 activity is associated with *Cux1*, we overexpressed the full length *Cux1* gene in either PRDM15 deleted or wildtype HSCs and performed a colony forming assay. Since CUX1 protein undergoes proteolytic processing to produce multiple isoforms with distinct functions^42^, we used the full-length p200 CUX1 construct, acknowledging that it will undergo proteolytic processing in cells. Our aim was to assess the overall CUX1 activity regardless of which isoform mediates the effect. After infection with lentiviral vector containing *Cux1* gene and selecting for GFP positive cells, HSCs were plated into methylcellulose plates and colonies were counted after 12–14 days, as described previously^43^ (Fig. 5d). As expected, PRDM15 deleted HSCs formed less colonies compared to wildtype HSCs (Fig. 5e), while Cux1 overexpression, which was verified by qPCR (**Supplementary Fig. 5c**), partially rescued colony formation in PRDM15 deleted HCSs. The partial rescue is likely due to the fact that PRDM15 regulates the expression of multiple target genes known to play a role in hematopoietic cell activity (e.g. *Wwox*^38,39^, *Cited2*^40,41^), ultimately, demonstrating that PRDM15 activity in HSCs is at least partially driven by regulation of *Cux1* expression.

## DISCUSSION

Our study identifies PRDM15 as an essential transcriptional regulator of HSC differentiation and survival, particularly under proliferative stress conditions such as bone marrow reconstitution. While PRDM15 appears dispensable in steady-state hematopoiesis, confirming its dispensability in adult mice^24^, proliferative demands during transplantation stress reveal its crucial role in maintaining effective lineage commitment and differentiation.

Mechanistically, PRDM15 regulates key metabolic and differentiation pathways essential for proper hematopoietic regeneration. Notably, PRDM15 maintains insulin signaling and key lipid metabolic pathways, crucial to sustaining stem cell differentiation and proliferation. Disruption of these pathways likely contributes to the differentiation blockade observed in PRDM15-deficient cells.

Beyond its metabolic role, PRDM15 directly regulates transcriptional programs that control quiescence and self-renewal. The identification of *Cux1* as a direct transcriptional target of PRDM15 establishes a novel regulatory axis linking PRDM15 to control of quiescence and proliferation in stem cells. Downregulation of *Cux1* following PRDM15 deletion may partially account for observed differentiation defects. Notably, *Cux1* knockout in the mice leads to transient hematopoeitic expansion, followed by reduction of HSCs and ultimately bone marrow failure. *Cux1* deletion lead to HSC exit from quiescence and HSC exhaustion^32^. In contrast, PRDM15 deletion results in reduced, but not abolished, *Cux1* expression producing a milder phenotype. This partial downregulation likely preserves limited Cux1 activity, mitigating the extent of hematopoietic exhaustion.

Furthermore, *Cux1* overexpression only partially rescued colony-forming potential after PRDM15 deletion, implying that additional downstream targets contribute to PRDM15-mediated regulation of hematopoiesis. Future studies should explore these targets to better define PRDM15’s broader regulatory network. For example, *Cited2* may represent a key mediator, as its activity influences HSC metabolism by promoting a shift from glycolysis to oxidative phosphorylation, which is associated with HSC exit from quiescence.^44^

Interestingly, CUX1 expression is also decreased in PRDM15-deficient B cell lymphoma models, where loss of PRDM15 impairs lymphomagenesis^24^. This shared regulation suggests that CUX1 acts as a downstream effector of PRDM15, linking its transcriptional control in both normal hematopoiesis and oncogenic contexts.

Clinically, these findings highlight the potential implications of PRDM15 dysfunction in situations demanding rapid hematopoietic recovery, such as post-chemotherapy or post-transplantation settings. PRDM15 modulation may offer therapeutic avenues for enhancing stem cell resilience and differentiation during hematopoietic stress.

In conclusion, our work underscores PRDM15’s role as a key transcription regulator, orchestrating stem cell differentiation and survival under proliferative stress, offering novel therapeutic potential for hematopoietic regeneration and disease management.

## METHODS

### Animal models and genotyping

The Prdm15^fl/fl^;R26^cre/cre^ mice have been described in detail (Mzoughi, Nature Genetics 2017). To develop PRDM15 FF vav-cre+ animals, Prdm15^fl/fl^;R26^cre/cre^ was bred to remove *R26*^*cre/cre*^. The resulting animals were bred with *B6.Cg-Commd10*^*Tg(Vav1-icre)A2Kio*^*/J* (Jax: 008610) to develop Prdm15^fl/fl^;Vav^cre^ animals. CD45.1 (B6.SJL-Ptprca Pepcb/BoyJ) and CD45.1/CD45.2 were bred in house.

To genotype, tails were digested in lysis buffer (100 mM sodium chloride, 25 mM EDTA, 10 mM Tris buffer (pH 8.0), 0.5% SDS, 0.2 mg/ml Proteinase K). Following incubation at 56 °C overnight, samples were heated at 98°C for 10 minutes and resuspended in nuclease free water.

100 ng of template DNA, 500 nM primers, and DreamTaq Green PCR master mix (Thermo Scientific #K10820) were combined for PCR. All primers are listed in **Supplementary Table S8**. The following cycling conditions were used: 95 °C for 5 min; 34 cycles of 95 °C for 45 s, 60 °C for 30 s and 72 °C for 40 s; 72 °C for 5 min.

Whole blood was collected by submandibular bleeds. Bleeds were performed every 4 weeks harvesting a maximum of 10% of circulating blood volume.

For PRDM15^fl/fl^;R26^cre/cre^ experiments, 1.5mg of tamoxifen was administered intraperitoneally in adult mice (6–8weeks) for 3 consecutive days. R26^cre/cre^ was used as control for PRDM15^fl/fl^;R26^cre/cre^ and was injected with the same dose as the experimental mice.

### Bone Marrow and Spleen Single Cell Isolation

Bone marrow was harvested by isolating the femur and the tibia. The femur and tibia are disconnected by removing the connecting cartilage. The epiphysis is moved to flush bone marrow from the medullary cavity of the femur and tibia. A 10CC syringe filled with cold DMEM with 10% FBS, 1% P/S, 1% HEPES, 1% non-essential amino acids, 1% sodium pyruvate and 0.143mM 2- β -mercaptoethanol, is used to flush bone marrow cells out of long bones. Resulting bone marrow was gently homogenized through a 70uM filter. The cells were incubated in red cell lysis buffer for 30 seconds. Cells are resuspended depending on final analysis or if cells will be used for transplantation.

Spleen single cell isolation was performed by homogenizing the spleen into a 70uM cell filter with a plunger into cold PBS. After initial spin, cells were incubated in red cell lysis buffer for 30 seconds. The cells are filtered a second time with a 40uM cell filter. After the final spin, cells are resuspended depending on final analysis.

### Recombination PCR

Recombination PCR. To confirm the deletion of the Prdm15 floxed exon (exon 4) following activation of Cre-recombinase, genomic DNA from cells was isolated using the Qiagen DNeasy blood & Tissue kit (cat. No. 69506). DNA was isolated from mouse tissues according to the genotyping protocol described above. The following primers were used for amplification:

Fwd-AAGACATTGGGTGCACAG, Rev-GGCTTCTGGGGTTCACTTT. The following cycling conditions were used for PCRs: an initial incubation at 95 °C for 5 min, followed by 36 cycles of denaturation (45s at 95°C), annealing (30s at 57°C), elongation (2 min at 72 °C), and a terminal incubation at 72 °C for 7 min.

### KIT isolation

Single cell bone marrow suspension cells were sorted into c-Kit+ and c-Kit- populations with Miltenyi Biotec Beads. CD117 MicroBeads (Catalog Number – 130-097-146) were incubated with bone marrow cells for 15 minutes at 4°C. Spin the cells and resuspend in PBS Buffer (PBS, 0.5% FBS, 2mM EDTA). Placed Miltenyi columns in appropriate magnetic field and transferred resuspended cells into Miltenyi columns. Washed with PBS buffer until negative fraction clears columns. Removed column from magnetic field and flushed positive cells out of Miltenyi columns with PBS buffer and plunger.

### Mouse Bone Marrow Transplant

CD45.1 animals (B6.SJL-Ptprca Pepcb/BoyJ) were pretreated with neomycin at a dose of 1mg/ml in drinking water, 1 week prior to irradiation and transplant. Animals were irradiated twice within 3–4 hours at 550 cGy and 500 cGy with a total radiation dose of 1050 cGy. 24 hours after the first dose of radiation, 5E6 of total bone marrow cells in 200ul of PBS are transplanted retro orbitally. Animals are anesthetized with isoflurane. Irradiated animals continued to be treated with neomycin for 2 weeks post-transplant, providing fresh neomycin every 2 days to ensure antibiotics are not degraded. Experimental analysis was performed after 1 month post-transplant to allow for engraftment.

For competition studies, CD45.1 animals were crossed to CD45.2 B6 animals to produce CD45.1/CD45.2 heterozygotes. CD45.1/CD45.2 animals were irradiated at age 10–12 weeks old. Mice were irradiated as described above. 2.5E6 of CD45.1 WT bone marrow cells were harvested at 6–8 weeks old and combined with 2.5E6 of Prdm15^fl/fl^ Vav^cre^ or Vav^cre^ bone marrow cells. Cells were injected retro-orbitally. Experimental analysis was performed 1 month after transplantation to allow for engraftment. Blood samples were collected at weeks 4, 8, 12 and 16.

### Flow cytometry of whole blood, bone marrow and spleen

For whole blood immunostaining, 20ul of blood whole blood were used and stained with antibody cocktail resuspended in FACS buffer (PBS, 2% FBS, 1mM EDTA). After 30 minutes of staining, blood is resuspended in eBioscience 1-step Fix/Lyse Solution (Catalog Number - 00-5333-54) for 30 minutes. Whole blood and solution were spun at 700g for 5 mins and resuspended in FACS buffer for flow cytometry acquisition. To determine absolute number of cells, Precision Count Beads (Catalog Number – 424902) were added and the published equation was used to calculate total number of cells.

Single cell bone marrow and spleen samples were stained with antibody cocktail resuspended in FACS buffer for 30 minutes. The following antibodies were used:

Monoclonal anti-CD11b, SuperBright 436, clone M1/70, Invitrogen, Cat# 62-0112-82

Monoclonal anti-CD8, BV510, clone 53-6.7, Biolegend, Cat# 100751

Monoclonal anti-CD19, BV570, clone 6D5, Biolegend, Cat# 115535

Monoclonal anti- I-A/I-E, BV605, clone M5/114.15.2, Biolegend, Cat# 107639

Monoclonal anti-NK1.1, BV650, clone PK136, Biolegend, Cat# 108736

Monoclonal anti-CD45, BV750, clone 30-F11, Biolegend, Cat# 103157

Monoclonal anti-CD3, Alexa Fluor 532, clone 17A2, Invitrogen, Cat# 58-0032-82

Monoclonal anti-CD4, PerCP, clone GK1.5, Biolegend, Cat# 100432

Monoclonal anti-CD11c, PerCP/Cyanine 5.5, clone N418, Biolegend, Cat# 117328

Monoclonal anti-NKp46, PE/Dazzle 594, clone 29A1.4, Biolegend, Cat# 137630

Monoclonal anti-Ly-6G, PE/Cyanine 7, clone 1A8, Biolegend, Cat # 127618

Monoclonal anti-Ki67, Alexa Fluor 700, clone B56, BD, Cat# 561277

Monoclonal anti-CD45.2, BUV661, clone 104, BD, Cat# 741516

Monoclonal anti-CD135, BV421, clone A2F10, Biolegend, Cat# 135315

Monoclonal anti-CD117, BV650, clone 2B8, Biolegend, Cat# 105853

Monoclonal anti-CD16/32. BV711, clone 93, Biolegend, Cat# 101337

Monoclonal anti-CD45.1, PerCP-Cy5.5, clone A20, Biolegend, Cat# 110727

Monoclonal anti-CD34, PE, clone HM34, Biolegend, Cat# 128609

Monoclonal anti-CD217, APC, clone PAJ-17R, Invitrogen, Cat# 17-7182-82

Monoclonal anti-Ly6A/E, BUV395, Clone D7, Invitrogen, Cat# 363-5981-82

LIVE/DEAD Fixable Dead Cell Stain Kit, Invitrogen, Cat# L23105

Centrifuge the cells after 30 minutes and incubate with 4% paraformaldehyde (PFA) for 30 minutes in room temperature. Wash the cells with FACS buffer and resuspend fixed cells in FACS buffer for acquisition. Stained samples were acquired with AuroraCS. Antibodies were titrated with splenocytes prior to experiment.

### RNAseq

At least 500,000 cells snap-frozen bone marrow cells were used for the RNA sequencing protocol. The cells were lysed and homogenized with Trizol. The lysate was incubated and centrifuged with chloroform. After centrifuge separation, transferred aqueous upper layer into a new tube and bind RNA by adding one volume of 70% ethanol. Transferred mixture into Spin Cartridges from PureLink RNA MiniKit (Invitrogen), spin and wash the column. Performed DNase treatment by adding DNase mixture to column. After incubation columns were washed per PureLink RNA MiniKit protocol. The RNA were quantified with Qubit RNA High Sensitivity Assay (Invitrogen) and quality was determined by Agilent Tape Station. Only RNA samples with RNA Integrity Number equivalent (RIN^e^) scores greater than 7 were used for further sequencing and analysis.

Library preparation was per- formed following the TruSeq RNA sample preparation v2 guide (Illumina).

Raw sequencing read quality was assessed using FastQC v0.11.9. Adapter trimming and removal of low-quality bases were performed with Trim Galore v0.6.6. Trimmed reads were aligned to the mouse genome GRCm39 (GENCODE v34) using the STAR aligner v2.7.10a in solo/single-cell mode, following guidelines from the Deplancke Lab GitHub repository. Gene-level count tables were generated in R using the Matrix v1.6–5 and tidyverse v2.0.0 packages and served as input for differential expression analysis with DESeq2 v1.42.1. Genes were considered differentially expressed when the adjusted *p*-value < 0.05 and the absolute log₂ fold change > 0.5. Gene Set Enrichment Analysis (GSEA) was performed using the fgsea v1.28.0 package in R, ranking genes by the DESeq2 Wald statistic and testing against the GO:BP library (Gene Ontology Consortium, 2023). Plots were generated using ggplot2 v3.5.2.

### ChIPseq

All steps of ChIP experiments were carried at 4 °C in the presence of protease inhibitors, unless stated otherwise. In brief, 10 million cells were fixed in 1% formaldehyde for 10 min at RT. The reaction was quenched by adding glycine to a final concentration of 0.125 M. The cells were washed in PBS, harvested in SDS lysis buffer and frozen at −80 °C overnight. The next day, the cells were pelleted by centrifugation, resuspended in ice-cold IP buffer and sonicated for 10 cycles of (30 s ON/60 s OFF) with Diagenode Bioruptor Homogenizer to obtain chromatin fragments of ~300–800 bp. The lysates were precleared for 4 h in a 1:1 A/G Dynabeads mix (blocked in 5 mg/ml lipid-free BSA). The beads were removed by centrifugation and samples were incubated with PRDM15 antibody with rotation overnight at 4 °C. The next day, pre-washed dynabeads were added, and the samples were incubated with rotation for 4h at 4°C. The beads were then pelleted by centrifugation and washed. The immunoprecipitated DNA was eluted in 1% SDS and 0.1 M NaHCO3, and the samples were de- crosslinked overnight at 65 °C. The eluted material was purified with MinElute PCR purification Kit (Qiagen 28804), and the DNA was resuspended in T- buffer (10 mM Tris–HCl, pH 8). ChIP qPCR was used to validate results.

### ChIP qPCR

Cux1 Primers:

Forward: GGCCGCCATCTTGAGACATA

Reverse: TCAGGCTTCGGACTCCATG

Spry1 Primers: (Positive Control)

Forward: GGCAATCGACTTTTGTTAGAGCGT

Reverse: AAACCCCTGCGTGCTGAGCACT

Cad Primers: (Negative Control)

Forward: GGCTGCTTGCGCCGT

Reverse: CAGAGCCCTAAGCGTAGTGAGC

### ChIP sequencing and bioinformatics:

Raw sequencing read quality was assessed using FastQC v0.11.9. Adapter trimming and removal of low-quality bases were performed using Trim Galore v0.6.6. Trimmed reads were aligned to the mouse reference genome GRCm39 (Gencode M34) using Bowtie2 v2.4.4 with default parameters. Read alignment quality was assessed using FastQC and aligned reads were filtered to retain those with MAPQ ≥20, excluding mitochondrial reads and those mapping to unplaced or random contigs, using SAMtools v1.11. PCR duplicates were removed using Picard v3.1.1. bamCoverage from deepTools v3.2.1 was used to generate normalized coverage tracks with parameters –normalizeUsingRPKM –binsize 10. Peak calling was performed using MACS3 v3.0.2 with a q-value threshold of 0.05, selected after evaluating multiple cutoffs. Peaks were called for both treatment and input samples against an IgG control. To identify treatment-specific binding events, peaks overlapping with input-derived peaks were removed. Resulting peaks were filtered against ENCODE blacklist regions for mm39 using BEDTools v2.31.0. Genomic annotation of peak regions was performed using ChIPseeker with UCSC mm39 knownGene annotations. Motif enrichment was performed using HOMER v4.10 (findMotifsGenome.pl) with default parameters. For *ckit−*, the analysis was restricted to the top 200 peaks ranked by fold enrichment. For *ckit+*, all identified peaks were used without further subsetting. Gene Ontology (GO) enrichment analysis was carried out using the clusterProfiler 4.10.1 R package with the org.Mm.eg.db database, focusing on Biological Process terms. Results were filtered at an adjusted *p*-value < 0.05 using the Benjamini–Hochberg correction.

### Rescue Experiments with CUX1 Overexpression

Rescue experiments performed with bone marrow cells from PRDM15^fl/fl^ Vav^cre^ or Vav^cre^ sorted for LKS cells. LKS cells were plated at 150,000–200,000 cells per mL in SFEM-medium with thrombopoietic (100ng/mL), SCF (100ng/mL), Flt3 ligand (50ng/mL), IL7 (50ng/mL) and 1% penicillin/streptomycin. After overnight incubation, cells were plated in RetroNectin coated plate and infected with lentivirus containing Cux1 overexpression plasmid. Plasmid procured from VectorBuilder with full length Cux1 (NM_001291233.2) overexpression controlled by EF1alpha promoter and an EGFP marker. 293T were transfected to produce lentivirus containing plasmids. Cells infected with lentivirus were sorted by GFP expression and plated in MethoCult (*M3434*) at 5,000 cells per 3 mL split into duplicates. Colonies counted after 12–14 days. Colonies were counted by 3 separate individuals to ensure colony count trends are consistent. CUX1 overexpression was verified by qPCR (Primers listed on **Supplementary Table S8**) of methylcellulose colonies.

### PRDM15 Knockout in Human HSPCs

Fetal liver CD34+ HSPCs were sorted and cultured 96 well round-bottom plates (VWR) for 48 hours in serum-free X-VIVO 10 medium (Lonza) supplemented with 1 % bovine serum albumin fraction V (Roche), 1x L-glutamine (Thermo Fisher), 1x penicillin-streptomycin (Thermo Fisher) and the following cytokines (Biotechne): FLT3 Ligand (100 ng/mL), G-CSF (10 ng/mL), SCF (100 ng/mL), TPO (15 ng/mL) and IL-6 (10 ng/mL)(here you can cite our recent preprint paper: PMID: 40166161). CRISPR/Cas9 RNP electroporations were performed using chemically synthesized gRNAs (IDT), recombinant Cas9 nuclease (IDT), and the 4D-Nucleofector (Lonza). For the preparation of the RNP complex, Alt-R CRISPR/Cas9 crRNA and tracrRNA (IDT) were reconstituted to a concentration of 200 μM in TE Buffer (IDT). The crRNA and tracrRNA were then mixed in a 1:1 ratio and annealed using a thermocycler at 95° C for 5 min, followed by cooling to room temperature. For each electroporation reaction, 1.2 μl of crRNA:tracrRNA complex, 1.7 μl of Cas9 protein, and 2.1 μl of PBS (Cytiva) were combined in a low-binding Eppendorf tube and incubated at room temperature for 15 min to form the complex. After incubation,1 μl of 100 μM electroporation enhancer (IDT) was added to the mixture.

Pre-cultured cells were washed with pre-warmed PBS and centrifuged at 350 × g for 10 min. Approximately 2.5×10^5^ cells were resuspended in 20 μl of Buffer P3 (Lonza) per reaction and added to the tube containing the CRISPR/Cas9 gRNA RNP complex. The mixture was thoroughly mixed by pipetting and then transferred to the electroporation chamber (Lonza). The cells were electroporated using the 4D-Nucleofector with the program DZ-100. Immediately following electroporation, 180 μl of pre-warmed X-VIVO 10 medium (as described above) was added to the electroporation chamber, and cells were transferred to a 96-well round-bottom plate. The electroporated cells were allowed to recover overnight at 37° C in a tissue culture incubator. Then, cells were transplanted into sublethally irradiated NSG mice via intrafemoral injection. 10 weeks post-transplantation, the mice were euthanized, and bone marrow cells were isolated. Human cells engraftment, different human cell populations were subsequently analyzed by flow cytometry. Engraftment was considered positive if the human CD45+ engraftment level in the bone marrow was greater than 1%, with the population presenting as dense clusters rather than dispersed signals.

PRDM15 gRNA

gRNA-1: CCCAGAAAACAGCGCCCCCG

gRNA-2: CTCATGTCTTTTGAAGCTGC

Control (OR2W5) gRNA gRNA-1: GACAACCAGGAGGACGCACT

gRNA-2: CTCCCGGTGTGGACGTCGCA

genotyping primers

PRDM15-F: ACAGTCACACGACCGTATCAG

PRDM15-R: TGGAAGGGGCTGTCAAGTAG

OR2W5-F: TCGGCCTGGACTGGAGAAAA

OR2W5-R: GAGACCACTGTGAGGTGAGA

Antibodies used in this in vivo experiment:

Mouse monoclonal anti-CD45, V500, clone HI30, BD, Cat#560777

Mouse monoclonal anti-CD3, FITC, clone SK7, BD, Cat#349201

Mouse monoclonal anti-CD19, PE-Cy7, clone HIB19, BD, Cat#560728

Mouse monoclonal anti-CD33, BV711, clone WM53,BD. Cat#563171

Mouse monoclonal anti-CD34, APC-Cy7, clone 581,BD, Custom conjugation

Mouse monoclonal anti-CD117, APC, clone 104D2, BD, Cat#333233

Mouse monoclonal anti-CD41, PE-Cy5, clone P2, Beckman Coulter, Cat#6607116

Mouse monoclonal anti-GlyA, PE, clone KC16, Beckman Coulter, Cat# IM2211U

Mouse monoclonal anti-CD71, BV650, clone L01.1, BD, Cat#745273

## Supplementary Material

Supplementary Files

This is a list of supplementary files associated with this preprint. Click to download.

- SupplementalTableNJPSubmitted.xlsx
